# Genome Sequence of *Bacillus anthracis* Larissa, Associated with a Case of Cutaneous Anthrax in Greece

**DOI:** 10.1128/genomeA.01273-15

**Published:** 2015-11-12

**Authors:** Gregor Grass, Matthias Hanczaruk, Markus Antwerpen

**Affiliations:** Bundeswehr Institute of Microbiology, Munich, Germany

## Abstract

We report the genome sequence of *Bacillus anthracis* strain Larissa, isolated from a diseased sheep associated with a human case of cutaneous anthrax in Central Greece from 2012. Genome sequence analysis of strain Larissa may aid in describing phylogenetic relationships of *B. anthracis* isolates in Southeastern European countries.

## GENOME ANNOUNCEMENT

Today, the zoonotic disease anthrax is rare in many European countries, including Greece. However, in July of 2012, a stockbreeder from the village of Tsabournia (prefecture Larissa, Thessaly) in Central Greece acquired cutaneous anthrax after slaughtering and flaying one of his sheep. The patient went successfully through antibiotic therapy with penicillin G; however, shortly after, two more sheep and two dogs fed with contaminated viscera of the butchered sheep succumbed to the disease (1).

Biological samples from the patient did not yield live *Bacillus anthracis*, but those from the diseased sheep did (1). Characterization of this isolate termed Larissa revealed the presence of both virulence plasmids (pXO1 and pXO2) in accordance with its virulent phenotype. Genotyping placed the bacterium within the widespread Trans-Eurasian (TEA, A.Br.008/011, [2]) clade of *B. anthracis*, which is typical for large areas of Europe and Asia.

Nowadays, outbreak events caused by *B. anthracis* are all but absent from Greece; however, endospores of *B. anthracis* can remain dormant in soil for many decades. The genome sequence of isolate Larissa thus also provides a small glimpse into the past on the diversity of the bacterium. Further genetic comparisons with isolates from neighboring countries might provide further insights into the geographic relationships of this notorious pathogen.

Whole-genome shotgun (WGS) sequencing of *B. anthracis* Larissa was performed by Ion Torrent sequencing technology (Ion Torrent Systems Inc., USA). For the WGS library, 561,905 reads with a total of 120 Mb were generated. Using Bowtie2 software (3), 93.01% of the reads were mapped to the Ames Ancestor chromosome, plasmids pXO1 and pXO2 (NC_007530.2, NC_007322.2, AE017335.3). The G+C content was calculated using an in-house Python script. Unmapped reads were assembled using the Velvet algorithm (4), and resulting contigs were analyzed. No significant hits were found after blasting against the NR nucleotide database of NCBI.

The total length of the genome shotgun sequence of *B. anthracis* Larissa was 5,227,401 bp with a coverage of 22-fold, and the mean G+C content was 33.8%. The plasmids pXO1 and pXO2 yielded sizes of 181,658 and 94,752 bp, with coverages of 49- and 25-fold, respectively. The transfer utility of Geneious software version 7.01 (Biomatters) was used for annotation.

The genome encodes 5,542 putative coding sequences. In the final genome sequence, 11 copies of the 16S rRNA, the 5S rRNAs, and the 23S rRNA were identified, as well as 95 tRNAs loci.

### Nucleotide sequence accession numbers.

This genome project has been deposited at DDBJ/EMBL/GenBank under the accession numbers CP012519, CP012520, and CP012521. The versions described in this paper are the ﬁrst versions.
